# Comparative assessment of the copy number of satellite repeats in the genome of Triticeae species

**DOI:** 10.18699/VJGB-23-109

**Published:** 2023-12

**Authors:** P.Yu. Kroupin, A.I. Yurkina, A.A. Kocheshkova, D.S. Ulyanov, G.I. Karlov, M.G. Divashuk

**Affiliations:** All-Russia Research Institute of Agricultural Biotechnology, Moscow, Russia; All-Russia Research Institute of Agricultural Biotechnology, Moscow, Russia; All-Russia Research Institute of Agricultural Biotechnology, Moscow, Russia; All-Russia Research Institute of Agricultural Biotechnology, Moscow, Russia; All-Russia Research Institute of Agricultural Biotechnology, Moscow, Russia; All-Russia Research Institute of Agricultural Biotechnology, Moscow, Russia

**Keywords:** Triticeae, satellite repeats, qPCR, whole-genome sequencing, Triticeae, сателлитные повторы, qPCR, полногеномное секвенирование

## Abstract

Satellite repeats are a significant component of the genome of Triticeae and play a crucial role in the speciation. They are a valuable tool for studying these processes. Pseudoroegneria species play a special role among grasses, as they are considered putative donors of the St-genome in many polyploid species. The aim of this study was to compare the copy number of satellite repeats in the genomes of Triticeae species. Quantitative real-time PCR was applied to determine the copy numbers of 22 newly discovered satellite repeats revealed in the whole-genome sequences of Pseudoroegneria species and one additional repeat previously identified in the genome of Aegilops crassa. The study focused on seven species of Pseudoroegneria, three species of Thinopyrum, Elymus pendulinus, Ae. tauschii, Secale cereale, and Triticum aestivum. Based on the copy number level and coefficients of variation, we identified three groups of repeats: those with low variability between species (medium-copy CL82), those with medium variability (low- and medium-copy CL67, CL3, CL185, CL119, CL192, CL89, CL115, CL95, CL168), and those with high coefficients of variation (CL190, CL184, CL300, CL128, CL207, CL69, CL220, CL101, CL262, CL186, CL134, CL251, CL244). CL69 exhibited a specific high copy number in all Pseudoroegneria species, while CL101 was found in both Pseudoroegneria and Th. junceum, CL244 in Th. bessarabicum, CL184 in P. cognata and S. cereale. CL95, CL128, CL168, CL186, CL207, and CL300 exhibited higher copy numbers in P. cognata compared to other species; CL3, CL95, CL115, CL119, CL190, CL220, CL207, and CL300 in P. kosaninii; CL89 in P. libanotica; CL134 in P. geniculata. Our assessment of the copy number of new satellite repeats in the St-genome and the analysis of their amplification specificity between species can contribute to the molecular-genetic and chromosome markers used for evolutionary, phylogenetic, and population studies of Triticeae species

## Abstract (ru)

Сателлитные повторы составляют значительную часть генома Пшеницевых, играя важную роль в видообразовании, что делает их ценным инструментом для изучения этих процессов. Особое место среди злаков занимают виды Pseudoroegneria – наиболее вероятные доноры St-генома у многих полиплоидов. Цель настоящего исследования состояла в сравнительной оценке копийности сателлитных повторов в геномах Triticeae. С помощью количественной полимеразной цепной реакции в реальном времени была установлена копийность 22 сателлитных повторов, выявленных в полногеномных нуклеотидных последовательностях видов Pseudoroegneria, и одного ранее опубликованного повтора, обнаруженного в геноме Aegilops crassa. Объектами анализа стали семь видов Pseudoroegneria, три вида Thinopyrum, Elymus pendulinus, Ae. tauschii, Secale cereale и Triticum aestivum. По уровню копийности и коэффициентам вариации нами выделено три группы повторов: с низким уровнем вариативности между видами (среднекопийный CL82), средним уровнем вариатив- ности (низко- и среднекопийные CL67, CL3, CL185, CL119, CL192, CL89, CL115, CL95, CL168) и с высокими значениями коэффициента вариации (CL190, CL184, CL300, CL128, CL207, CL69, CL220, CL101, CL262, CL186, CL134, CL251, CL244). Повтор CL69 показал специфическую высокую копийность для всех видов Pseudoroegneria, CL101 – у Pseudoroegneria и Th. junceum, CL244 – у Th. bessarabicum, CL184 – у P. cognata и S. cereale. У P. cognata более высокую копийность, по сравнению с остальными видами, проявили повторы CL95, CL128, CL168, CL186, CL207, CL300; у P. kosaninii – CL3, CL95, CL115, CL119, CL190, CL220, CL207 и CL300; у P. libanotica – CL89; у P. geniculata – CL134. Проведенные нами оценка копийности сателлитных повторов, найденных в St-геноме, и анализ специфичности их амплификации между видами могут пополнить арсенал молекулярно-генетических и цитогенетических маркеров, используемых для эволюционных, филогенетических и популяционных иссле- дований представителей трибы Пшеницевых.

## Introduction

Triticeae is an economically important tribe of the Poaceae family, comprising approximately 500 species of annual and perennial herbaceous plants (NCBI database: https://www. ncbi.nlm.nih.gov/Taxonomy/Browser/wwwtax.cgi). Wheat, rye, barley, and fodder grasses are among the species of this tribe that play a significant role in providing food for humanity and have also become an integral part of animal diets (Hodkinson, 2018).

The interest in studying phylogenetic relationships within the Triticeae tribe is largely driven by the potential of wild wheat species to serve as valuable sources of economically important genes for the improvement of cultivated cereals. For example, Thinopyrum and Dasypyrum serve as gene donors for resistance to various diseases (Yang et al., 2005; Luo P.G. et al., 2009; Salina et al., 2015; Wang S. et al., 2019; Li L.F. et al., 2022; Guo et al., 2023). By crossing wheat and Agropyron, it is possible to increase head productivity (Zhang J. et al., 2016). Representatives of Pseudoroegneria are droughtresistant and are used as pasture grasses (Wu et al., 2023b).

The Triticeae tribe consists of approximately 100 annual and 400 perennial species, which carry one (in diploids) or several (in polyploids) of 13 genomes (Wang, Lu, 2014). Representatives of Pseudoroegneria carry the St-genome, and it is believed that this genus was the donor of the St-genome for Elymus and some Thinopyrum species (Mahelka et al., 2011; Dobryakova, 2017; Linc et al., 2017; Lei et al., 2018; Chen N. et al., 2020; Agafonov et al., 2021). Plants of the genus Agropyron, at all ploidy levels (2n = 2x/4x/6x), are distinguished by the presence of a P-genome (Zhang Y. et al., 2015). Genome J (=E) is mainly composed of diploids Thinopyrum bessarabicum (genome J = Jb) and Th. elongatum (genome E = Je). The J-genome is evolutionarily close to the D-subgenome of common wheat, and the most likely donor of the D-subgenome is Aegilops tauschii (Baker et al., 2020). This may explain why the chromosomes of the D-subgenome in the introgressive lines of common wheat, developed with the aim of improving it through hybridization with Thinopyrum, show the highest frequency of introgressions from the J-genome (Chen Q. et al., 2001; Liu Z. et al., 2007; Cui et al., 2018).

At present, the origin, relationships, and proximity of genomes within the Triticeae tribe remain controversial and uncertain. The challenges associated with studying Triticeae genomes are primarily due to the significant differences between the polyploid subgenome and the ancestral genome of the diploid parent organism. These differences arise from the modifications that occur during evolution. Additionally, the diploid ancestor of the donor organism may have become extinct or has not yet been found (Jakob, Blattner, 2010; Liu Q.-L. et al., 2020; Sha et al., 2022). Perennial polyploid species, for example, Th. intermedium and Th. ponticum, may have an unbalanced genome or chromosomal translocations (Kruppa, Molnar-Lang, 2016; Liu Y. et al., 2023). This could be associated with the transition to vegetative reproduction, which does not involve sexual processes for seed formation and therefore does not require stable meiosis (Comai et al., 2005; Husband et al., 2013). The same species are characterized by recombinant subgenomes, the origin of which is still unclear (Wang R.R.C. et al., 2015; Liu Y. et al., 2023). Discussions continue regarding potential donors of the Y- subgenomes in Elymus and Roegneria (Yan et al., 2011; Liu Q.-L. et al., 2020; Wu et al., 2021), as well as the maternal form in the occurrence of Thinopyrum, Roegneria, Elymus, Kengyilia, and other polyploids (Mahelka et al., 2011; Luo X. et al., 2012; Zeng et al., 2012; Lei et al., 2018; Chen N. et al., 2020)

In addition, there is a problem of correlation between the species identification of a particular specimen based on botanical traits (often influenced by the environment) and that based on molecular genetic and cytogenetic characteristics (Wang, Lu, 2014; Al-Saghir, 2016; Rodionov, 2022). For example, this issue arises in Elymus, as demonstrated by studies conducted by A.V. Rodionov et al. (2019), V. Lucia et al. (2019), and L. Tan et al. (2021). Another example is the relationship between Th. elongatum and Th. bessarabicum that bear fairly similar genomes, but differ in botanical characteristics (Grewal et al., 2018; Dai et al., 2021; Chen C. et al., 2023). The problem is compounded by the fact that natural spontaneous hybrids are often found (Chen C. et al., 2022; Luo Y.C. et al., 2022; Wu et al., 2023a). The study of phylogenetic relationships deepens our understanding of evolutionary processes in plants and speciation, and helps to improve biosystematics. The acquired knowledge will enhance the efficiency of utilizing genetic resources from wild species by improving the understanding of their proximity to the genomes of cultivated crops and the potential for obtaining valuable introgressions.

The genome of Triticeae grasses is characterized by a large size, which complicates whole-genome deep sequencing and makes assembly difficult (Rabanus-Wallace, Stein, 2019). A significant portion of the Triticeae genome is composed of repetitive DNA, known as repeats. The repeatome include mobile elements, gene clusters (specifically the 5S and 45S rRNA genes), and satellite repeats (Dvořák, 2009; Shcherban et al., 2015; Gao et al., 2023; Vershinin et al., 2023).

Satellite repeats are tandem repeating non-coding sequences that exist as arrays of varying lengths in genetically silent heterochromatin regions (Badaeva, Salina, 2013). Satellite repeats are considered to be the most variable and rapidly evolving components. They can be species-specific or common to closely related species (Belyayev et al., 2019; Garrido- Ramos, 2021; Thakur et al., 2021). Comparative analysis of copy number, nucleotide sequences, and localization on the chromosomes serves as a tool for basic phylogenetic and evolutionary studies of plants, including Triticeae species (Anamthawat-Jónsson, Heslop-Harrison, 1993; Vershinin et al., 1994; Kishii et al., 1999; Anamthawat-Jónsson et al., 2009; Han et al., 2017; Linc et al., 2017; Ruban, Badaeva, 2018; Said et al., 2018; Salina, Adonina, 2019; Dai et al., 2021; Wu et al., 2021; Chen C. et al., 2022; Kroupin et al., 2023; Shi et al., 2023). Satellite repeats have found practical use as PCR markers and chromosomal markers for identifying introgressions of alien genetic material containing valuable economic traits in the genomes of cultivated cereals (Li G. et al., 2016; Han et al., 2017; Liu L. et al., 2018; Chen J. et al., 2019).

Tangible progress has been made in the study of Triticeae genomes, owing to the invention of whole-genome sequencing methods and bioinformatic algorithms for analyzing the data obtained (Rabanus-Wallace, Stein, 2019; Gao et al., 2023). The rapid growth in the volume of information on genomewide sequences of Triticeae has significantly accelerated and simplified the search for repetitive DNA that can be used as chromosomal markers (Du et al., 2017; Said et al., 2018; Tang et al., 2018; Chen J. et al., 2019; Kroupin et al., 2019a, 2022; Lang et al., 2019a; Liu Q.-L. et al., 2020; Wu et al., 2021, 2022). Due to the significant presence of repetitive DNA in the Triticeae genomes, information about satellite repeats can be obtained even through sequencing with low coverage. This greatly simplifies the process of searching for repeated sequences (Navajas-Perez, Paterson, 2009; Kroupin et al., 2019b; Šatovic-Vukšic, Plohl, 2023).

A well-established method for quantifying the number of copies of repetitive DNA, including satellite repeats, is quantitative real-time PCR (qPCR) (Harpke, Peterson, 2007; Navajas-Pérez et al., 2009; Baruch, Kashkush, 2012; Feliciello et al., 2015; Divashuk et al., 2016, 2019, 2022; Pereiera et al., 2018; Shams, Raskina, 2018). Compared to Southern blot or dot-blot hybridization on a membrane, or fluorescent in situ hybridization on chromosomes, qPCR has proven to be an easier-to-use, accurate, and effective method for assessing the copy number of the target sequence. This method allows researchers to identify the number of copies of satellite repeats in the genome and the variability between genomes (Kalendar et al., 2020; Pös et al., 2021).

In this study, the whole-genome sequencing of Pseudoroegneria spicata, P. libanotica, P. tauri, P. geniculata, P. cognata, and P. kosaninii revealed the presence of 22 satellite repeats. In order to comprehend the potential of using them as tools for evolutionary and phylogenetic studies of wild representatives of the Triticeae tribe, as well as for studying wide hybrids using molecular biology and cytogenetics methods, it is crucial to first determine the copy number of satellite repeats in the genomes of St-genome carriers. Therefore, we have chosen Pseudoroegneria species with different ploidy levels as our research subject.

To assess the specificity of satellite repeats for the St- genome, we included Thinopyrum species in the experiment, which contain the J-genome that is common among Triticeae. We also selected Th. intermedium with the JrJvsSt-genomic formula, serving as a carrier of the St-subgenome and Stspecific repeats in the recombinant Jvs-genome. To explore the potential of utilizing the identified satellite repeats for the characterization of distant wheat and rye hybrids, Triticum aestivum and Secale cereale accessions were included in the study. In addition, due to the evolutionary proximity of the J- and D-genomes, we included the Ae. tauschii accession. E. pendulinus was also used, carrying both the St-subgenome targeted by our work and the Y-subgenome of unknown origin, which is common among Elymus sensu lato species. The experiment also included a satellite repeat of CL244, which we obtained as a result of analyzing the whole-genome nucleotide sequence of Ae. crassa (D1Xcr), a carrier of the D-genome variant (Kroupin et al., 2022). Despite this, CL244 was not found in Ae. tauschii, showed a small number of hybridization sites on the chromosomes of T. aestivum and Ae. crassa, while on the chromosomes of the Jb-genome in Th. bessarabicum, bright signals were observed indicating a high level of its abundance.

## Materials and methods

The Triticeae species with various genomic compositions, as listed in the Table, served as the material for our study.

**Table 1.**
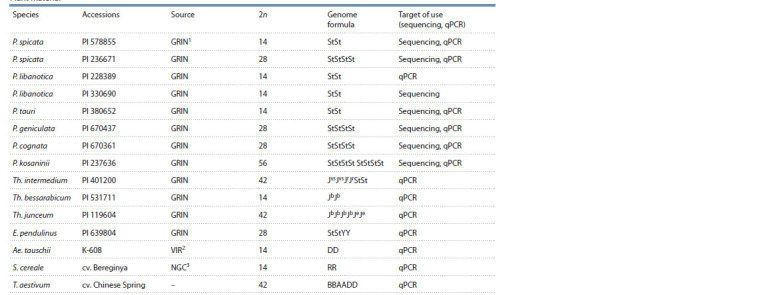
Plant material 1 GRIN – germplasm resources information network of the Agricultural Research Service of the US Department of Agriculture (USDA-ARS Germplasm Resources Information Network). 2 VIR – N.I. Vavilov All-Russian Institute of Plant Genetic Resources. 3 NGC – P.P. Lukyanenko National Grain Center.

The young leaves of the plants were frozen in liquid nitrogen. Genomic DNA was then isolated using the CTAB protocol (Rogers, Bendich, 1985). This DNA was used for subsequent sequencing and quantitative PCR (qPCR). The concentration and purity of the isolated DNA were tested using Qubit 4 (Thermo Fisher Scientific, USA) and electrophoresis in a 0.8 % agarose gel.

Shotgun sequencing libraries were synthesized using the Swift 2S Turbo DNA Library Kit (Swift Bioscience, USA) in accordance with the manufacturer’s protocol. To assess the quality of the libraries, a test run was conducted on the MiSeq device (Illumina, Inc., USA). The libraries were then converted and sequenced using DNBSEQ-G400 on one track. The initial amount of DNA was 25 ng. The fragments were approximately 350 bp long and were indexed at both ends using the Swift 2S Turbo Unique Dual Indexing Kit (Swift Bioscience). Sequencing was performed on Illumina NextSeq (Illumina, Inc.), using the NextSeq 500/550 Mid Output Kit v.2.5 (Illumina, Inc.).

Bioinformatic analysis was conducted on the processing and assembly of the reads of nucleotide sequences that involved a sequence of satellite tandem repeats. The uniqueness of these sequences was evaluated in comparison to previously published methods described in P.Y. Kroupin et al. (2022). The primer sequences for the identified monomers of satellite repeats are provided in Supplementary Material 11.


Supplementary Materials are available in the online version of the paper: https://vavilov.elpub.ru/jour/manager/files/Suppl_Kroupin_Engl_27_8.xlsx


Quantitative real-time PCR was conducted using DNA from the accessions listed in the Table as a template, with three replicates. The amplification was carried out using a CFX realtime amplifier system (Bio-Rad Laboratories, Inc., USA) and a reaction mixture of Real-Time PCR Mix containing the Eva Green fluorophore (Synthol Ltd., Russia) in accordance with the manufacturer’s protocol. A single-copy gene, VRN1, was used as the reference gene. Primer concentration in mixtures consisted of 10 ng/μl, while DNA concentration was 0.4 ng/ μl. Amplification was performed according to the following program: preincubation – 10 min at 95 °С; followed by 40 cycles: denaturation – 10 s at 95 °С; primer annealing – 30 s at 60 °С.

Statistical analysis, including the calculation of the average values of Cq, standard deviation, and the corresponding number of copies relative to the reference gene VRN1, was performed using Bio-Rad CFX and Manager 3.1 software. To assess the similarity of copy numbers among repeats, we have introduced the concept of “repeat copy number pattern”, a set of copy number values for a specific repeat within the set of species being studied. To assess the similarity of copy number among the species under investigation, we have introduced the concept of “species copy number pattern”, a set of values of the copy number of the satellite repeats being studied for a particular species

Pearson’s correlation coefficients (r) were calculated using Statistica 12 software (StatSoft, USA) to determine the relationship between repeat copy number patterns and species copy number patterns. The significance level was set at p <0.05. Diagrams were constructed using the principal component analysis method for satellite repeats and the studied species. The diagrams were based on the data obtained from Statistica 12 software, which included information on the copy number of satellite repeats. The coefficient of variation of the satellite repeatability values between species was calculated using Microsoft Excel (USA).

## Results

Characteristics of identified satellite repeats

In the framework of the present study, a total of 22 satellite repeats were found in separate genome sequence assemblies. As a result of analyzing the nucleotide sequence of the diploid accession of the P. spicata (2n = 14) genome, 10 repeats were identified. These repeats include CL69, CL82, CL101, CL119, CL128, CL168, CL184, CL207, CL251, and CL262. Additionally, four repeats were found in the nucleotide sequence of the P. tauri genome (CL67, CL89, CL185, and CL192), as well as in the P. kosaninii genome (CL3, CL115, CL220, and CL300). Furthermore, one repeat was found in each of the genomes of P. libanotica (CL95), P. geniculata (CL134), P. cognata (CL186), and a tetraploid P. spicata (CL190).

The characteristics of the identified satellite including its length and the most similar sequences in the NCBI database are presented in Supplementary Material 1. After comparing the nucleotide sequences of the 22 repeats with those previously published in the NCBI, we did not find any matches for nine of them (CL69, CL89, CL95, CL168, CL185, CL207, CL251, CL262, CL300). For the remaining 13, the level of identity among similar published sequences ranged from 70 to 98 %. This indicates that they are different from previously published sequences (see Supplementary Material 1).

Two satellite repeats showed similarities to repeats found in common wheat: CL119 was similar to the pTa-465 clone (77 % identity), and CL101 was similar to the Spelt1-like subtelomeric repeat (80 % identity). Three repeats showed similarities to the following known satellites: CL220 to CL219, which was detected in the Ae. crassa genome (82 %), CL134 to CL97 from the Th. bessarabicum genome (71 %) and CL186 to ACRI_TR_CL80 from the A. cristatum genome (70 %). The other three repeats show similarities to microsatellites: CL128 has similaritiy to L15 identified in the P. stipifolia genome (84 %), CL190 shows similarity to P523 from the genome of Ae. tauschii (81 %), and CL82, to pTa-451 from common wheat (88 %). Four repeats we found showed similarity to the following mobile elements: CL184, which has a 98 % similarity to the Cassandra retrotransposon from the rye genome, CL67 and CL115, which have a 91 and 78 % similarity to retrotransposons from the barley genome Cereba and Sandra5, respectively, and CL192, which has a similarity to transposon XJ from the Ae. tauschii genome (70 %). The CL3 repeat was most similar to the E-gene-specific marker Th. elongatum 51-6 (79 %).

Assessment of the copy number of satellite repeats using qPCR

The data obtained on the relative copy numbers of 23 satellite repeats in 14 species, calculated in relation to the reference single-copy VRN1 gene, are shown in Supplementary Material 2.1. All of the repeats we studied differed in terms of copy numbers and the coefficient of variation between the species. Since the order of the obtained copy numbers varied significantly, the results were presented in the form of a decimal logarithm for the convenience of comparing their abundance (see the Figure and Supplementary Material 2.2). Hereafter, we will simply refer to the decimal logarithm of relative copy number as “copy number”. Since the copy number rate varied from 0 to 5, the repetitions were classified into the following groups based on their copy number: low (≤ 2), medium (> 2, < 4), and high (≥ 4). Since the coefficient of variation ranged from 0 to 0.6, we assumed that the variability was low when its value was less than 0.1, medium when it fell between 0.1 and 0.25, and high when it exceeded 0.25.

**Fig. 1.**
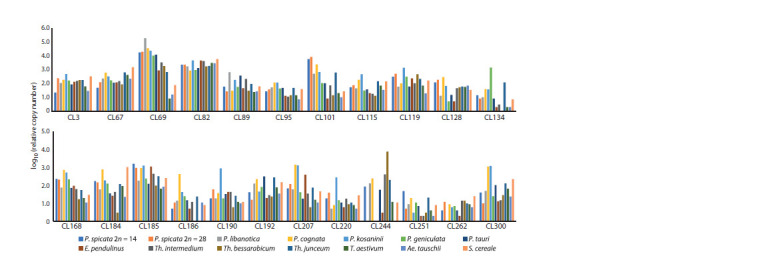
Relative copy number of the satellite repeats in the studied species of the Triticeae tribe expressed as a decimal logarithm.

CL82 turned out to be the least variable repeat: its abundance was medium-closer to high and ranged from 2.9 to 3.8.

The medium coefficient of variation for the copy number of satellite repeats in all studied accessions (from 0.16 to 0.25) was observed in nine specific repeats, namely CL67, CL3, CL185, CL119, CL192, CL89, CL115, CL95, and CL168 (listed in ascending order based on their coefficient of variation). The average copy number values in Pseudoroegneria species were 2–11 % higher compared to the entire studied collection. However, CL67 had the highest copy number in the rye genome (3.2). CL89 showed the highest value in P. libanotica (2.8), CL3, CL119, and CL115 had the highest values in P. kosaninii (2.7, 3.1, and 2.7, respectively), CL95 had the highest value in both P. cognata and P. kosaninii (2.1), and CL168 had the highest value in P. cognata (2.9). The remaining repeats of this group were generally characterized by a low-to-medium level of copy number across all accessions. The minimum copy number was observed in CL192, ranging from 1.2 to 2.5, while the maximum copy number was found in CL185, ranging from 1.8 to 3.2.

A high level of variability was observed in the following 13 repeats: CL190, CL184, CL300, CL128, CL207, CL69, CL220, CL101, CL262, CL186, CL134, CL251, and CL244. The coefficient of variation ranged from 0.27 to 0.43. In this group, the following repeats can be distinguished: CL69 with a high copy number in Pseudoroegneria species (4.0–5.3), medium in Thinopyrum and E. pendulinus (2.8–3.0), and low in the rest; CL101 with a medium copy number in Pseudoroegneria (2.0–3.9) and Th. junceum (2.8), low in the rest; CL244, close-to-high in Th. bessarabicum (3.9), significantly varies in Pseudoroegneria species (0–2.4), and is medium to low in the others. CL184 is the highest in P. cognata (2.9) and S. cereale (3.0) compared to the others (0.5–2.3). Individual repeats showed the highest copy number in specific Pseudoroegneria species: CL128 and CL186 in P. cognata (2.5 and 2.6, respectively), CL190 and CL220 in P. kosaninii (2.5 and 3.0, respectively), CL134 in P. geniculata (3.1), CL207 and CL300 in P. cognata and P. kosaninii (ranging from 3.1 to 3.2). CL251 and CL262 were characterized by an overall low copy rate, ranging between 0.3–1.7 and 0–1.4, respectively.

Correlation analysis (see Supplementary Material 2.3) and principal component analysis (see Supplementary Material 2.4) were used to identify the following groups with similar repeat copy number patterns: 1) CL3, CL115, CL119, CL190, and CL220 (r ≥ 0.77); 2) CL95, CL207, and CL300 (r ≥ 0.87); 3) CL128, CL168, and CL186 (r ≥ 0.72). Correlation analysis (see Supplementary Material 2.5) and principal component analysis (see Supplementary Material 2.6) revealed a high level of similarity in the species copy number patterns among the following groups: 1) Pseudoroegneria accessions (r > 0.9); 2) E. pendulinus, Th. intermedium and Th. junceum (r > 0.8); 3) rye, common wheat, E. pendulinus, Th. junceum, and Ae. tauschii (r ≥ 0.89). The medium level of similarity in the species copy number pattern was observed between Th. intermedium and Pseudoroegneria (r > 0.6). The species copy number pattern in Th. bessarabicum, on average, showed the least similarity with other species.

## Discussion

Satellite repeats constitute a significant portion of the Triticeae genome and play a crucial role in the formation and evolution of new species. As a consequence, they serve as valuable tools for analyzing these processes (Shcherban, 2015; Salina, Adonina, 2019; Vershinin et al., 2023). The search for new satellite repeats is necessary to understand the phylogenetic relationships and evolution of the Triticeae tribe, which is of significant importance to humans. One of the initial steps in determining the suitability of the identified satellite repeats as tools for such studies is to conduct a comparative assessment of their copy number in related species.

Some of the satellite repeats we found in the St-genome showed a similar copy number among the studied species. Homologs have been found in the genomes of wheat and barley, suggesting their common origin. CL82 and CL119 showed similarity to pTa-451 and pTa-465, respectively, which were identified in T. aestivum (Komura et al., 2013). CL67 is similar to the centromeric retrotransposon Cereba (Hudakova et al., 2001) and is conserved in Triticeae (Dvořák, 2009). Although CL3 is 79 % identical to the E-specific repeat 51-6, it did not show any specificity for Thinopyrum species in our study. This suggests that we have discovered an older and less genome-specific variant.

CL69 was distinguished by a high copy number in Pseudoroegneria accessions and a medium copy number in Thinopyrum and E. pendulinus species. This may indicate its occurrence before the divergence of the St- and J-genomes. CL101 has a medium copy number in Pseudoroegneria and Th. junceum species and could also occur in a common ancestor of the St- and J-genomes. Since CL101 is 80 % identical to the subtelomeric Spelt1-like repeat, it is likely that it may have a common origin with Spelt-1, which is common in Triticum and Aegilops. This repeat is characterized by significant variation in copy number between species (Pestsova et al., 1998; Ruban, Badaeva, 2018). The copy number of CL69 and CL101 in individual accessions is relatively high, reaching values of up to 3.9 and 5.3, respectively. This makes them suitable candidates for use as chromosomal markers in the FISH procedure. Further experiments using the FISH method will show whether these repeats can serve as chromosomal markers for identifying the St-subgenome in polyploid species, such as E. pendulinus, studying recombinant Jvs-genomes in intermediate wheatgrass and investigating chromosomal rearrangements in wide wheat hybrids.

The highest copy number value in P. cognata and S. cereale was demonstrated by CL184, which shows similarity to the Cassandra retrotransposon found in the rye genome. Cassandra is found in the genomes of many plant species and is characterized by significant differences in copy number between them (Kalendar et al., 2020). Since one of the mechanisms by which satellite repeats are propagated throughout the genome is through the movement of retroelements (Garrido- Ramos, 2021; Šatović-Vukšić, Plohl, 2023), it is possible that we have identified a repeat that has been retained as a consequence of Cassandra spreading along the ancestral lineage of the St- and R-genomes

CL244, previously identified by us in the Ae. crassa genome, was characterized by a higher copy number in Th. bessarabicum than in common wheat, Ae. crassa and Ae. tauschii (Kroupin et al., 2022). In this study, the results were confirmed. However, at the same time, there was a significant variation in copy number between Pseudoreogneria species, which could be attributed to the elimination or accumulation of CL244 during speciation and subsequent evolution. CL244 has terminal localization on chromosome Th. bessarabicum (Kroupin et al., 2022), and can presumably accumulate or be eliminated in various species, similar to the terminal repeats of Spelt-1 and Spelt-52 in Aegilops and Triticum (Raskina et al., 2011; Ruban, Badaeva, 2018) or pSc200 and pSc250 in rye (Evtushenko et al., 2016).

CL220, which is specific to P. kosaninii, exhibited similarities with CL219, which we had previously identified in the Ae. crassa genome (Kroupin et al., 2022). CL186, specific to P. cognata, showed similarity to ACRI_CL80, which was identified in A. cristatum (P-genome) (Said et al., 2018). Both repeats probably arose before the divergence of Triticeae genomes from a common ancestor and accumulated in separate species at certain periods. Since CL219 and ACRI_CL80 were localized on separate chromosomes, it can be inferred that CL220 and CL186 will also exhibit chromosome-specific localization on the chromosomes of P. kosaninii and P. cognata, respectively.

We have identified repeats that vary in copy number between Pseudoroegneria species with varying levels of ploidy. For example, the octaploid P. kosaninii is characterized by a high copy number of CL115, CL190, and CL220, while the tetraploid P. geniculata has a high copy number of CL134. The observed differences in the abundance of the repeats may be attributed to polyploidization processes. This is because tandem repeats in the centromeric and terminal regions have a significant impact on chromosome recognition and divergence during cell division. This is particularly relevant for homeological genomes in polyploid plants (March, 2019; Aguilar, Prieto, 2021). Such subgenome- and even chromosome-specific repetitive elements have been detected in polyploid species such as wheat, oats, and intermedium wheatgrass. These elements are apparently necessary for the differentiation of subgenomes during cell division (Shrama, Raina, 2005; Liu Z. et al., 2008; Divashuk et al., 2016; Lang et al., 2019b; Su et al., 2019).

Comparison of the repeat copy number patterns helped determine which of them have similar copy numbers among the studied accessions (see Supplementary Materials 2.3 and 2.4). CL3, CL115, CL119, CL190, and CL220 were grouped together because they exhibited the highest levels of copy number in P. kosaninii. CL95, CL207, and CL300 are more specific to P. cognata and P. kosaninii. In CL128, CL168, and CL186, the maximum copy number was observed in P. cognata. A comparison of the species copy number patterns allowed for a general understanding of which accessions are characterized by similar repeat copy numbers

The overall clustering of Pseudoroegneria species (see Supplementary Materials 2.5 and 2.6) indicates that, in general, they exhibit similar copy numbers of repeats that are different from those of other species. The copy number pattern of E. pendulinus (2n = 28, StStYY) differed from that of Pseudoroegneria, suggesting that the St-specific repeats we discovered could be valuable for analyzing the St-subgenome of E. pendulinus and other Elymus sensu lato accessions. Thinopyrum and common wheat exhibited different copy number patterns. CL244 and CL69 can be utilized to identify wheatwheatgrass hybrids and detect introgressions from all three studied wheatgrass species into the wheat genome. Similarly, CL134 and CL251 can be used for this purpose in Th. jun-ceum.

L. Wang et al. (2017) found a repeat of St2-80 in the genome of P. libanotica, hybridizing along the entire length of the St- (sub)genome chromosomes and only in the terminal regions of the E-, H-, P-, and Y-(sub)genomes. Q.-L. Liu et al. (2020) identified mobile elements in the genome of P. stipifolia, including dispersed repeats S13, S158, and S21, which showed varying intensity between the chromosomes of the St- and Y-subgenomes. They also found S5, which had a specific point localization and differed between the chromosomes of the St- and Y-subgenomes. D. Wu et al. (2022) created chromosomal markers STlib_96, STlib_98, and STlib_117 based on satellite repeats of P. libanotica. However, the possibility of using these markers for the analysis of allopolyploids with the St-genome is not reported.

Our assessment of the copy number of satellite repeats found in the St-genome and the determination of their amplification specificity between species can enhance the range of molecular genetic and cytogenetic markers utilized in studying the Triticeae tribe. The copy number of satellite repeats can vary significantly between species, populations, and even within them (Wang Q. et al., 2010; Belyaev, Raskina, 2013; Pollak et al., 2018; Tao et al., 2021). The satellite repeats identified in the present study can be useful, among other purposes, for population studies of Pseudoroegneria and Triticeae species.

## Conclusion

Based on the data from whole-genome sequencing of Pseudoroegneria accessions, we identified 22 satellite repeats. In the genomes of 14 representatives of the Triticeae tribe, we determined their copy number, including CL244, which was previously discovered in Ae. crassa. As a result of the evaluation, the studied repeats were classified according to the level of abundance and variability between species. The satellite repeats identified in the present study can be used as molecular genetic markers for evolutionary, phylogenetic, and population studies of Triticeae. They also have the potential to serve as cytogenetic markers for in situ hybridization.

## Conflict of interest

The authors declare no conflict of interest.
